# Letheon

**Published:** 1847-08

**Authors:** 


*Letheon,*—The Boston Medical and Surgical Journal states that the Legislature of Connecticut has recognised the claim of Dr. Horace Wells, of Hartford, as the sole discoverer of the (so called) Letheon, and passed him a vote of thanks.

